# Missing-Data Handling Methods for Lifelogs-Based Wellness Index Estimation: Comparative Analysis With Panel Data

**DOI:** 10.2196/20597

**Published:** 2020-12-17

**Authors:** Ki-Hun Kim, Kwang-Jae Kim

**Affiliations:** 1 Faculty of Industrial Design Engineering Delft University of Technology Delft Netherlands; 2 Department of Industrial Engineering Ulsan National Institute of Science and Technology Ulsan Republic of Korea; 3 Department of Industrial and Management Engineering Pohang University of Science and Technology Pohang Republic of Korea

**Keywords:** lifelogs-based wellness index, missing-data handling, health behavior lifelogs, panel data, smart wellness service

## Abstract

**Background:**

A lifelogs-based wellness index (LWI) is a function for calculating wellness scores based on health behavior lifelogs (eg, daily walking steps and sleep times collected via a smartwatch). A wellness score intuitively shows the users of smart wellness services the overall condition of their health behaviors. LWI development includes estimation (ie, estimating coefficients in LWI with data). A panel data set comprising health behavior lifelogs allows LWI estimation to control for unobserved variables, thereby resulting in less bias. However, these data sets typically have missing data due to events that occur in daily life (eg, smart devices stop collecting data when batteries are depleted), which can introduce biases into LWI coefficients. Thus, the appropriate choice of method to handle missing data is important for reducing biases in LWI estimations with panel data. However, there is a lack of research in this area.

**Objective:**

This study aims to identify a suitable missing-data handling method for LWI estimation with panel data.

**Methods:**

Listwise deletion, mean imputation, expectation maximization–based multiple imputation, predictive-mean matching–based multiple imputation, k-nearest neighbors–based imputation, and low-rank approximation–based imputation were comparatively evaluated by simulating an existing case of LWI development. A panel data set comprising health behavior lifelogs of 41 college students over 4 weeks was transformed into a reference data set without any missing data. Then, 200 simulated data sets were generated by randomly introducing missing data at proportions from 1% to 80%. The missing-data handling methods were each applied to transform the simulated data sets into complete data sets, and coefficients in a linear LWI were estimated for each complete data set. For each proportion for each method, a bias measure was calculated by comparing the estimated coefficient values with values estimated from the reference data set.

**Results:**

Methods performed differently depending on the proportion of missing data. For 1% to 30% proportions, low-rank approximation–based imputation, predictive-mean matching–based multiple imputation, and expectation maximization–based multiple imputation were superior. For 31% to 60% proportions, low-rank approximation–based imputation and predictive-mean matching–based multiple imputation performed best. For over 60% proportions, only low-rank approximation–based imputation performed acceptably.

**Conclusions:**

Low-rank approximation–based imputation was the best of the 6 data-handling methods regardless of the proportion of missing data. This superiority is generalizable to other panel data sets comprising health behavior lifelogs given their verified low-rank nature, for which low-rank approximation–based imputation is known to perform effectively. This result will guide missing-data handling in reducing coefficient biases in new development cases of linear LWIs with panel data.

## Introduction

### Background

Smart wellness services are designed to help individuals monitor their own wellness through smart devices, including smartphones and smartwatches [1]. Reports indicate that these services will see exponential growth alongside continued smart device penetration and the increasing size of the wellness market [2]. Their popularity is further evidenced by the high number of mobile health apps, with around 325,000 available in app stores in 2017 [3,4].

Smart wellness services can collect various health behavior lifelogs through the aid of smart devices [5]. For example, smartwatches, such as Fitbit, can record daily walking steps, total distances, and the number of sleeping hours [6], while smart patches, such as HealthPatch, can monitor heart rate, breathing rate, skin temperature, posture, number of walking steps, activity patterns, and sleep habits [7]. There are also devices for infants, such as Owlet smart socks, that send the child’s vital signs to their parents via smartphones, including information on heartrate, oxygen level, skin temperature, sleep quality, and sleeping position [8].

Existing smart wellness services utilize health behavior lifelogs to provide users with detailed records about health behaviors [9]. Fitbit provides a smart wellness service that primarily shows users detailed activity records (eg, daily walking steps), exercise habits (eg, type, time, and duration), sleep information (eg, start and end times), and dietary facts (eg, daily calorie intake). By focusing on the details of each health behavior, existing smart wellness services have a limitation in supporting users to easily identify their aggregate condition from multiple health behaviors. Users must synthesize the information, making it difficult to monitor overall progress.

A lifelogs-based wellness index (LWI), a function that transforms health behavior lifelogs into wellness scores for smart wellness service users, resolves this limitation [10]. The wellness scores quantitatively represent how well the user meets relevant recommended health behaviors. Such information, including a user’s current or past wellness scores, wellness score progress over time, and comparisons of their wellness scores [11], can be offered by smart wellness services. According to Platt et al [12], a wellness index is a critical feature of wellness apps for younger demographics. The utility of LWIs is thus expected to stimulate new LWI development .

An LWI can be developed through 3 key phases: definition, estimation, and assessment [10,11]. The definition phase refers to the selection of the LWI function type and a model for estimating the function that consists of behavior variables and a proxy variable as its independent variables and dependent variable, respectively. The behavior variables are potential constituents of an LWI, while the proxy variable is used in place of wellness scores, immeasurable during the development process. The estimation phase refers to the process of estimating the coefficients of the behavior variables in LWIs by collecting and preprocessing data, which are then fit with the estimation model. The assessment phase refers to the assessment of LWI generalizability and utility for users.

LWI estimation can lead to the reduction of coefficient biases through a panel data set of health behavior lifelogs. A panel data set follows a given sample of participants over time, thus providing multiple observations for each participant. Existing panel data analysis methods (eg, 1-way random effects regression) can only be applied to panel data sets. These methods can reduce biases in the coefficients by controlling for heterogeneity across participants, which is caused by unobserved variables [13].

A panel data set comprising health behavior lifelogs will likely contain large proportions of missing data. Such a data set is collected based on everyday user activities and is therefore exposed to various random events that result in missing data. For example, users may forget to wear smart devices or to record health behavior lifelogs, and the smart devices themselves will no longer record health behavior lifelogs when batteries are depleted. These random events often lead to large proportions of missing data. For example, missing data accounted for 18% of a panel data set in an LWI development case [10]. This rate was considered high considering that participants received reminders for the data collection.

Missing data can lead to 2 severe problems when attempting to estimate LWI coefficients. First, it can introduce biases to the coefficients [14,15]. This leads to low LWI generalizability for users. Second, most existing data analysis methods are only applicable to complete data sets (ie, data sets without missing data). Thus, incomplete data sets must be modified into complete ones [16]. A variety of missing data handling methods exist to address these problems, the choice of which becomes increasingly significant as the proportion of missing data increases [17]. However, few studies have identified which existing method is suitable for handling missing data in a panel data set that is composed of health behavior lifelogs.

This study identified a suitable method for LWI estimation with panel data based on an examination of 6 representative missing-data handling methods: listwise deletion, mean imputation, expectation maximization–based multiple imputation, predictive-mean matching–based multiple imputation, k-nearest neighbors–based imputation, and low-rank approximation–based imputation. These were selected from common missing-data handling methods from previous studies, specifically because they represented possible missing-data handling approaches in the context of LWI estimation.

The 6 abovementioned missing-data handling methods were comparatively evaluated for various missingness proportions of a panel data set by simulating an LWI development case originally presented by Kim et al [10]. The case estimated the coefficients in a linear LWI with a panel data set composed of health behavior lifelogs. Such cases are expected to become prevalent because linear functions help users understand how changes in each behavior variable influence their overall wellness scores [18]. This advantage of linear LWIs enables users to obtain 2 types of valuable insights. First, users can easily see which behavior variables substantially decrease or increase their wellness scores, thus motivating them to manage those variables. Second, users can create optimized plans for improving their wellness scores based on the relative effects of each behavior variable. Linear functions are also already prevalent in existing wellness-related indexes (eg, [10,19,20]).

### Missing-Data Handling Methods

**Figure 1:**
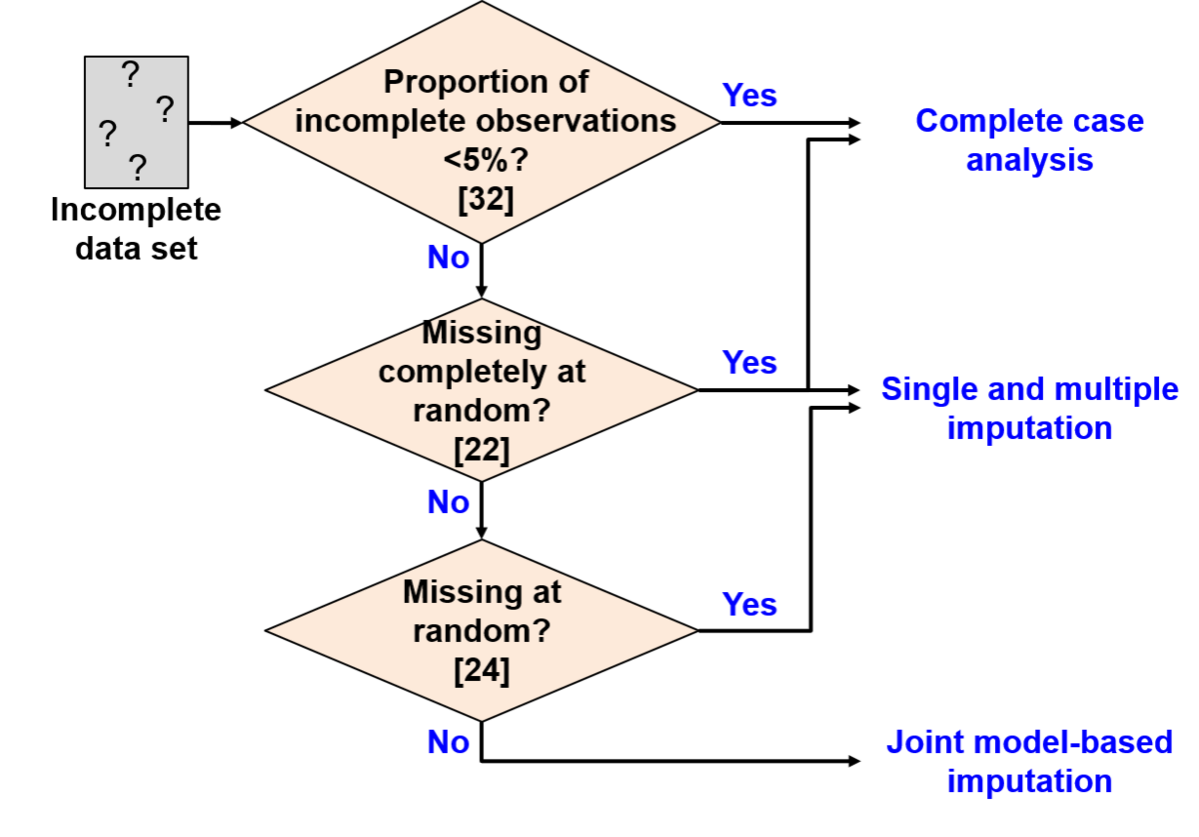
Existing recommendations for missing data handling.

Missing-data handling can be divided into 4 approaches, including complete case analysis, single imputation, multiple imputation, and joint model-based imputation (Figure 1). Complete case analysis excludes observations with missing values when analyzing data [21]. Single imputation produces only one complete data set by imputing missing values [22]. Multiple imputation creates multiple imputed data sets, applies a statistical analysis model to each one, and ultimately combines all analysis results to create an overall result [23]. Joint model-based imputation utilizes different distributions to model individuals with and without incomplete observations or directly models the relationship between the probability of a variable being missing and its missing value [24].

When selecting these 4 approaches, previous studies have used the missingness proportions and missingness mechanisms of data sets as major criteria for ensuring adequate selection for the data sets [25,26]. The missingness proportion is the ratio of the amount of missing values to the amount of missing and nonmissing values in the data set. The missingness mechanism can be divided into 3 types [14], including missing completely at random, missing at random, and missing not at random. First, missing completely at random is not related to any nonmissing or missing values in the data set. Second, missing at random entails that the missingness is independent of the missing values and is also conditional on nonmissing values. Third, the mechanism is missing not at random when the missingness depends on the missing values. As shown above, Figure 1 outlines the current recommendations for selecting adequate approaches based on both the missingness proportion and missingness mechanism.

A panel data set of health behavior lifelogs is likely to contain 5% or more of incomplete observations with a missingness mechanism similar to missing completely at random. This property is attributed to a variety of random daily events that result in missing data. For example, the LWI development case presented by Kim et al [10] showed an 18% proportion of incomplete observations even though participants received interventions reminding them about the need to collect data. Participants also reported that random daily events resulted in missing or abnormal data, specifically including issues such as forgetting to wear a smartwatch or not entering data via the smartphone app, depleted smartwatch batteries, and data transmission errors. Based on the flowchart shown in Figure 1, 3 of the missing-data handling approaches may be implemented for this property of a panel data set composed of health behavior lifelogs, including the complete case analysis, single imputation, and multiple imputation.

The 6 missing-data handling methods presented in Table 1 were selected to represent the complete case analysis, single imputation, and multiple imputation [21,27-31]. These methods are known to yield similar results given low missingness proportions (eg, less than 5% incomplete observations) [17,32]. The choice of missing-data handling method is known to become increasingly significant as the missingness proportion increases [17,32].

However, few previous studies have recommended which of the 6 missing-data handling methods are suitable for reducing coefficient biases according to the missingness proportion of a panel data set composed of health behavior lifelogs. This study filled that gap in the literature by comparatively evaluating the LWI coefficient biases of the 6 missing-data handling methods according to the missingness proportion of exactly such a panel data set.

**Table 1:** Representative missing-data handling methods applicable for LWI estimation.

Approach and method		Description
**Complete case analysis**		
	Listwise deletion [21]	Excludes all observations with missing values to conduct analysis
**Single imputation**		
	Mean imputation [21]	Imputes each missing value of a variable with the mean of observed values of the variable
	k-nearest neighbor–based imputation [30]	Imputes each missing value of a variable based on the observed values of the k-nearest neighbors
	Low-rank approximation–based imputation [29]	Predicts missing values as a linear combination of a small set of singular vectors
**Multiple imputation**		
	Expectation maximization–based multiple imputation [28]	Draws imputed values from the multivariate normal distribution of the data set estimated by expectation–maximization; multiple imputed data sets are estimated by repeating the imputation and separately analyzed; analysis results are pooled into the final result
	Predictive-mean matching–based multiple imputation [31]	Substitutes a missing value with a value randomly from complete observations, with regression-predicted values that are closest to the regression-predicted value for the missing value from the simulated regression model; multiple imputed data sets are estimated by repeating the imputation and separately analyzed; analysis results are pooled into the final result

## Methods

### Development Case: LWI for College Students

We previously developed an LWI for college students [10]. As a component of Onecare, a smart wellness service that supports individual-level health behavior monitoring for Korean college students based on their health behavior lifelogs, the index was developed to calculate daily wellness scores from lifelogs, thus intuitively showing users whether they were meeting recommended daily health behaviors. Daily wellness scores ranged from 0 to 100, indicating the worst and best conditions, respectively. The index was defined as a linear function consisting of 7 behavior variables (see Table 2), representing the critical health behaviors that Korean college students needed or wanted to manage. All such behaviors were identified based on expert interviews, target-user group discussions, and a literature review. As the daily wellness score was immeasurable during the development process, its proxy variable was also defined to estimate the index. More specifically, the proxy variable was the perceived score described in Table 2. Previous studies have regarded these types of perceived scores as valid measures for representing health. For example, patient-reported outcome measures are increasingly used in medical studies to represent psychometric self-evaluations of patient health [33,34].

**Table 2:** Variable descriptions.

Category and variable		Description (value meaning)
**Behavior variable**		
	Breakfast (or Lunch or Dinner)	Student’s self-rating of the day’s breakfast (or lunch or dinner) based on nutrition (0: skip, 33: low, 66: medium, 100: high)
	Exercise	Whether the student exercises or works out for more than 30 minutes during the day (0: no exercising, 100: exercising)
	Step achievement	Percentage indicating a ratio that the total number of walking steps in the day reached 10,000
	Sleep duration achievement	Percentage that the student’s sleep duration reached 7 hours between 6 PM of the previous day and 6 PM of the current day
	Golden time achievement	Percentage that the student slept during the golden time, which is 10 PM of the previous day to 2 AM of the current day
**Proxy variable**		
	Perceived score	Score that the student determines by evaluating overall condition of their critical health behaviors over the day

To establish an intuitive scoring system, all behavior variables and the proxy variable were set to range from 0 (worst) to 100 (best) [35]. Each variable was defined to minimize user participation in the data collection process. From this perspective, data on the 3 behavior variables (ie, golden time achievement, sleep duration achievement, and step achievement) were automatically collected by smartwatches worn by students. Students also could easily record data on the remaining 5 variables through a smartphone app.

A 1-way random effects regression model was used to estimate the index coefficients:



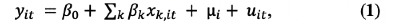



where *i*, *t*, and *k* denote the *i*th student, day *t*, and *k*th behavior variable, respectively; *y_it_* is the perceived score of the *i*th student on day *t*; β_0_ and β*_k_* are unknown coefficients; *x_k,it_* is the value of the *k*th behavior variable observed for the *i*th student on day *t*; μ*_i_* the unobserved student-specific random effect of the *i*th student, is independent and identically distributed, *N*(0, σ_μ_^2^), and is independent of *x_k,it_*; μ*_i_* controls for the effects of student-specific heterogeneity on *y_it_* and *u_it_*, the error term, is independent and identically distributed, *N*(0, σ*_u_*^2^).

This regression model was selected for 2 reasons. First, the index is a linear function. Second, the regression model was set to control for the unobserved student-specific random effects on the perceived score. Unobserved (or unmeasured) student-specific heterogeneity could exist in the regression model and thus influence the perceived score. For example, students may have different levels of interest in wellness, but these are unobserved in the regression model. However, those who are more interested in wellness may have higher standards for health behaviors, thus resulting in lower perceived scores. As the failure to control for such unobserved student-specific effects may produce misleading results [36], this was addressed by adding the effects to the regression model as μ*_i_*.

The data set used to estimate the regression model was compiled by collecting data on the daily life activities of 41 students including 21 undergraduate (15 males and 6 females) and 20 graduate students (15 males and 5 females), all of whom were attending a university in Korea. Their age statistics were as follows: average of 24.7, maximum of 30, minimum of 19, and a standard deviation of 2.8. A total of 1148 observations were thus collected over a 28-day period (November 3-30, 2015). An observation consisted of 1 student’s 1-day data for the 8 variables in the regression model.

Data preprocessing excluded the 264 observations including missing or abnormal values. Notably, students reported that these observations went through data collection problems (eg, forgetting to wear smartwatches, neglecting to enter data through the smartphone app, or depleting their smartwatch batteries). In this regard, they did not accurately reflect actual daily health behaviors of students. By excluding these observations, a panel data set comprised 884 complete observations from 41 students.

The LWI coefficients were estimated by fitting Eq (1) to the data set. Based on the estimated coefficients, the LWI was defined as a linear function consisting of the 7 following behavior variables: 0.151 × Breakfast + 0.163 × Lunch + 0.135 × Dinner + 0.135 × Exercise + 0.095 × Step achievement + 0.219 × Sleep duration achievement + 0.102 × Golden time achievement.

This study simulated the aforementioned LWI development case to evaluate biases regarding the regression coefficients that each of the 6 missing-data handling methods led to, as follows: the data set of the LWI development case was transformed into a reference data set that did not include any missing data; incomplete data sets were simulated by introducing missing data to the reference data set at various missingness proportions; the missing-data handling method changed all simulated data sets into complete data sets by handling their missing data; regression coefficients were estimated by fitting Eq (1) to the complete data sets; a bias measure of the missing-data handling method was calculated by comparing the estimated coefficient values with coefficient reference values. The coefficient reference values were estimated by fitting Eq (1) to the reference data set.

### Overview

In this study, we conducted a simulation to calculate a bias measure for incremental missingness proportions for each of the 6 methods. The bias measure was referred to as the grand-mean of absolute biases (GAB). For each missingness proportion, GAB was used to compare the coefficient biases, thus determining which missing-data handling methods was superior.

**Figure 2:**
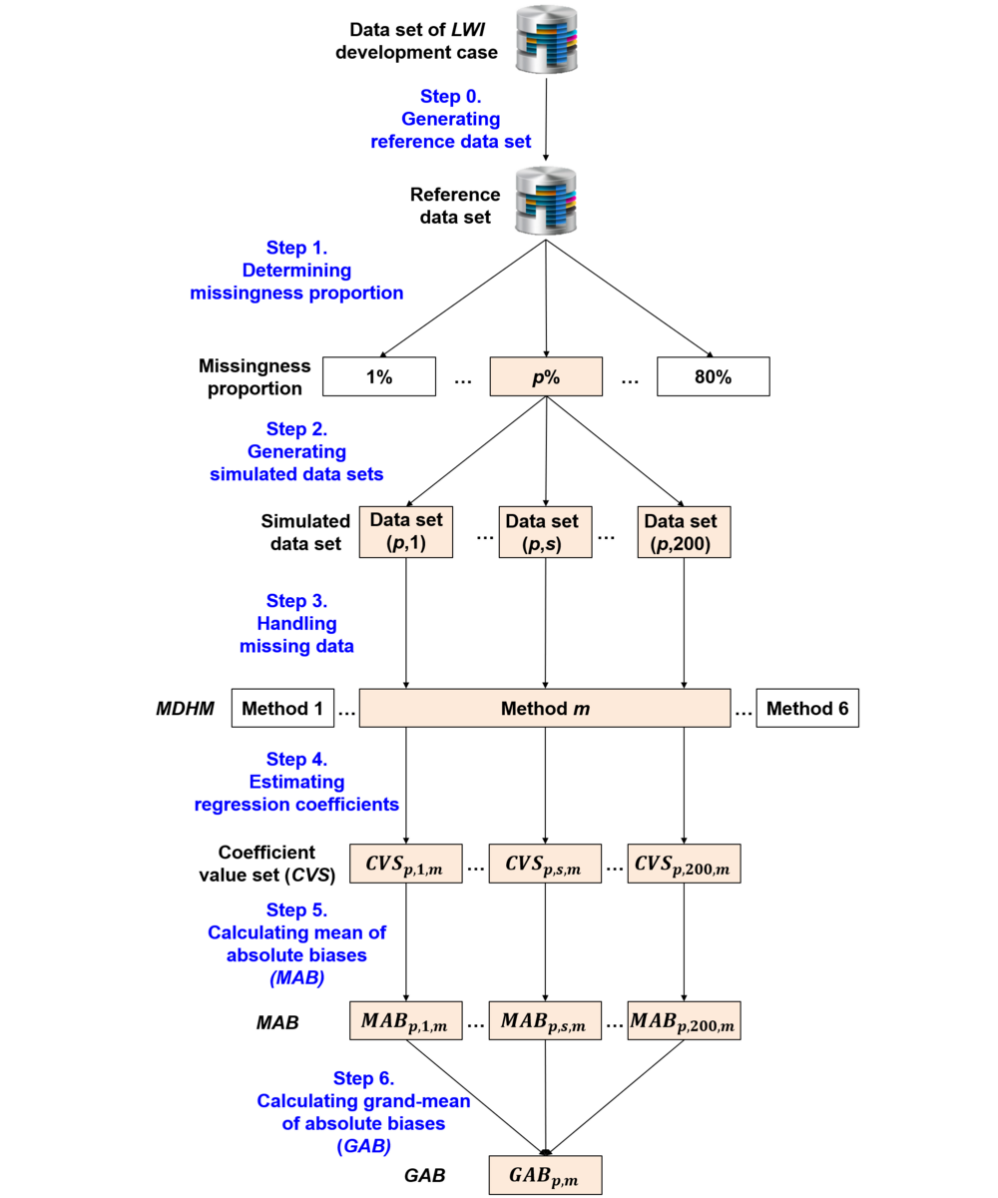
Research process.

Simulation steps are shown in Figure 2. In step 0, a reference data set was generated by transforming the data set from the development case. Steps 1 through 6 were then repeated for each missingness proportion, with each repetition calculating GAB for the 6 missing-data handling methods.

### Step 0: Generating the Reference Data Set

Step 0 was performed to generate a reference data set from the data set used in [10]. The reference data set included 884 observations of 41 students for 7 behavior variables and a perceived score variable. The descriptive statistics are provided in Table 3. Ranges of the variables were transformed from [*x*_min_, *x*_max_] to [*z*_min_=0, *z*_max_=1] using minimum-maximum normalization [37]:



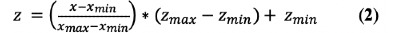



This normalization is generally recommended as preprocessing for data-mining algorithms, including missing-data handling methods [38].

**Table 3:** Descriptive statistics of the data set for developing the LWI for college students and regression results for the reference data set.

Variable	Descriptive statistics		Regression results	
	Mean (SD)	Range	Estimate (SE)	*P* value
Perceived score	63.4 (15.9)	0-100	N/A^a^	N/A
Breakfast	24.2 (36.2)	0-100	0.097 (0.014)	<.001
Lunch	63.5 (32.3)	0-100	0.105 (0.013)	<.001
Dinner	75.5 (27.5)	0-100	0.088 (0.015)	<.001
Exercise	5.3 (22.4)	0-100	0.087 (0.019)	<.001
Step achievement	74.6 (28.6)	0-100	0.061 (0.015)	<.001
Sleep duration achievement	86.0 (19.3)	6.7-100	0.131 (0.021)	<.001
Golden time achievement	14.2 (25.1)	0-100	0.066 (0.018)	<.001
(Intercept)	N/A	N/A	0.305 (0.029)	<.001

^a^N/A: not applicable.

The reference data set also included 40 dummy variables and a time variable. Here, the dummy variables coded the 41 students, while the value of time variable was determined based on the dates the data were collected, that is, between the first and last days of the data collection period (November 3-30, 2015):



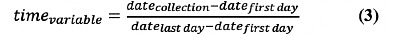



The resulting reference data set was 884×49 in dimension, as it contained all 884 observations mentioned above. Each observation included values for the 40 dummy variables, time variable, 7 behavior variables, and perceived score variable for a particular student on a given day. All variables ranged from 0 to 1.

### Step 1: Determining the Missingness Proportion

In Step 1, the missingness proportion was selected to evaluate the 6 missing-data handling methods. The missingness proportion increased from 1% to 80% by 1%. An increment of 1% was sufficiently small to observe how the performance of each method changed according to the missingness proportion. Previous studies [39-41] have used larger increments, for example, Hasan et al [39] used 4 levels (10%, 20%, 30%, and 40%), Marshall et al [40] used 5 levels (5%, 10%, 25%, 50%, and 75%), and Song et al [41] used 4 levels (10%, 15%, 20%, and 30%) of missingness proportion for simulations to evaluate method performance.

We used a range up to 80% because one method continued to show outstanding performance for proportion above 60% and a missingness proportion of 80% was too high to estimate coefficients with low biases. If a data set had such a high missingness proportion in practice, then it may be preferable to collect another data set instead of using data from the initial data set.

### Step 2: Generating the Simulated Data Sets

As shown in Figure 2, Step 2 generated 200 simulated data sets by randomly deleting the variable values from the reference data set according to missingness proportion *p*%. The random deletion implemented missing completely at random into the simulated data sets to reflect the missingness mechanism of a panel data set composed of health behavior lifelogs.

For proportion *p*%, there were many ways that missing data could be distributed across variables within the data set. Such a wide and varied distribution could affect missing-data handling method performance. However, there were too many possible missing data distributions to simulate all of them. Thus, this study randomly generated 200 simulated data sets for the missingness proportion, and then calculated the average of regression coefficient biases that each missing-data handling method produced across the 200 data sets. The average of each missing-data handling method was its performance measure (ie, GAB) for the missingness proportion. Similarly, Young and Johnson [42] had also calculated GABs of different missing-data handling methods across 200 simulated panel data sets in order to compare performance, although their work focused on multiple imputation and panel data sets related to family research.

### Step 3: Handling Missing Data

In Step 3, each of the 6 missing-data handling methods were applied to each of the 200 simulated data sets using R software (version 3.6.0). Listwise deletion and mean imputation were implemented by several lines of R code to automatically delete incomplete observations and substitute a missing value for a variable with the mean of its observed values, respectively. k-nearest neighbor–based imputation used the knnImputation function in the DMwR package [30]. The number of nearest neighbors was the odd value close to the squared root of complete observations in each simulated data set [43]. The package softImpute [29] was utilized as a low-rank approximation–based imputation. Its maximum rank and lambda were determined based on “warm starts [29].” Expectation maximization–based multiple imputation and predictive-mean matching–based multiple imputation used Amelia II [28] and MICE [31] packages, respectively. The number of multiple imputations was set to 5, based on published recommendations [44].

As a result of this step, each of the listwise deletion, mean imputation, k-nearest neighbor–based imputation, and low-rank approximation–based imputation methods resulted in a complete data set. For expectation maximization–based and predictive-mean matching–based multiple imputations, there were 5 complete data sets.

### Step 4: Estimating the Regression Coefficients

Eq (1) was fitted to each complete data set resulting from Step 3 using the plm package [45]. As a result, 8 coefficients (ie, β*_k_*) were estimated for each complete data set. Each listwise deletion, mean imputation, k-nearest neighbor–based imputation, and low-rank approximation–based imputation contained a set of the 8 coefficient values for a simulated data set because each one resulted in a compete data set for the simulated data set in Step 3. Each expectation maximization–based and predictive-mean matching–based multiple imputation contained 5 sets of the 8 coefficient values for a simulated data set, which were pooled into a single set each, following rules established by Rubin [14]. For each method, the set of 8 coefficient values was defined as coefficient value set *(CVS_p_*_,_*_s_*_,_*_m_*)={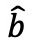
*_p_*_,_*_s_*_,_*_m_*_,0_,..., 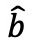
*_p_*_,_*_s_*_,_*_m_*_,7_}, where *CVS_p_*_,_*_s_*_,_*_m_* is the set of the 8 coefficient values that originated from the application of *m*th missing-data handling method to *s*th simulated data set of missing proportion *p*%; 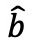
*_p_*_,_*_s_*_,_*_m_*_,k_ is *k*th coefficient value in *CVS_p_*_,_*_s_*_,_*_m_*; *p* ∈ {1%, 2%,..., 80%}; *s* ∈ {1, 2,…, 200}; and *m* ∈ {listwise deletion,..., predictive-mean matching–based multiple imputation}.

### Step 5: Calculating the Mean of Absolute Biases

Step 5 was performed to calculate a bias measure for each coefficient value set. Because a coefficient could have a certain amount of bias, each coefficient value set contained a total of 8 coefficient biases. The mean of absolute biases (MAB) was defined as a bias measure to calculate the average amount of the 8 coefficient biases for a given coefficient value set:



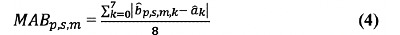



where 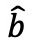
*_p_*_,_*_s_*_,_*_m_*_,_*_k_* ∈ *CVS_p_*_,_*_s_*_,_*_m_*; *â_k_* is the reference value of 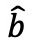
*_k_*; *â_k_* was estimated by fitting Eq (1) to the reference data set, as all simulated data sets were generated by deleting the missingness proportion *p*% of the reference data set. The estimate column in Table 3 provides the estimated values of *â_k_*. For missingness proportion *p*%, this step resulted in the 200 MABs of each missing-data handling method.

### Step 6: Calculating the GAB

We combined the 200 MABs for each method to create a bias measure that represented the average of its coefficient biases over the 200 simulated data sets of missingness proportion *p*%. By following Young and Johnson [42], the bias measure was defined as the GAB:



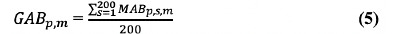



A low GAB indicated that the missing-data handling method led to small coefficient biases across the 200 simulated data sets of the missingness proportion. The GAB was used as the criterion for evaluating method performance.

## Results

**Figure 3:**
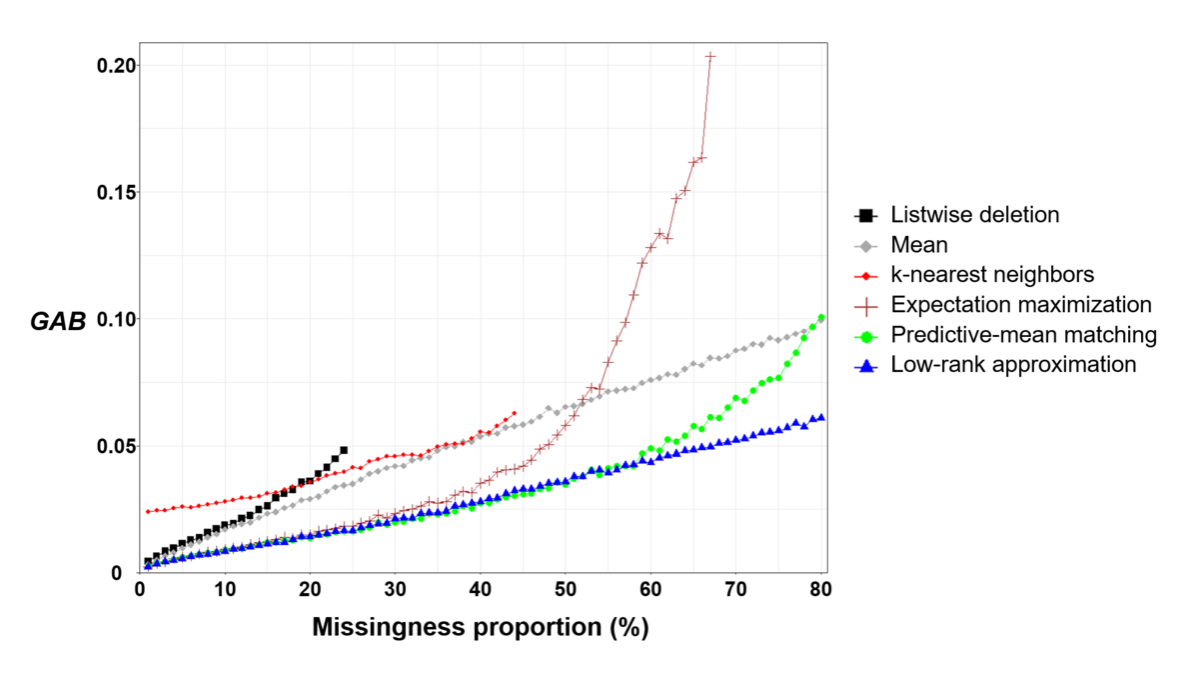
GAB results.

Figure 3 shows GABs for each missingness proportion. The listwise deletion, k-nearest neighbor–based imputation, and expectation maximization–based multiple imputation did not have GABs over missingness proportions of 24%, 44%, and 67%, respectively. Listwise deletion left too small number of complete observations to estimate the regression coefficients over missingness proportions of 24%. Both the k-nearest neighbor–based imputation and expectation maximization–based multiple imputation also failed to impute missing values over missingness proportions of 44% and 67%, respectively. The simulated data sets for these missingness proportions contained smaller numbers of complete observations than the minimum required for them to impute missing values.

**Figure 4:**
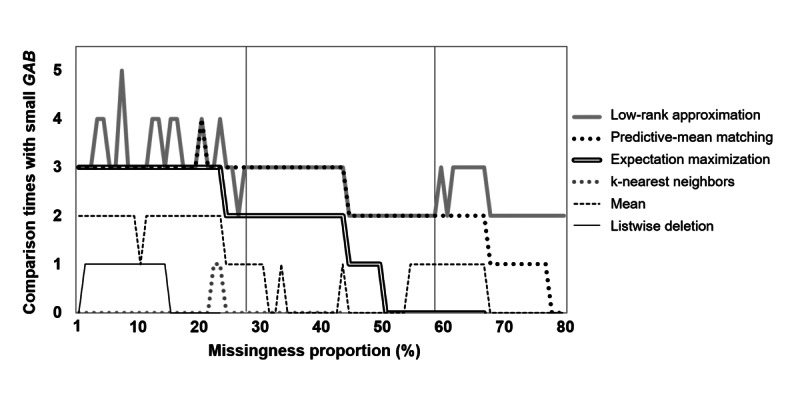
Number of pairwise comparisons with statistically small GAB differences.

Pairwise multiple comparison tests were conducted to statistically compare relative superiority among the 6 missing-data handling methods for each missingness proportion. The tests were conducted using Dunnett modified Tukey-Kramer pairwise multiple comparison at the .05 significance level [46]. Results provided the number of pairwise comparisons in which each missing-data handling method had statistically small GAB compared with all other missing-data handling methods for each missingness proportion. For interpretation purposes, a superior missing-data handling method will show the maximum number of pairwise comparisons with statistically small GAB (Figure 4). For example, the low-rank approximation–based imputation, predictive-mean matching–based multiple imputation, and expectation maximization–based multiple imputation were shown to be superior at a 1% missingness proportion (Figure 4).

Different missing-data handling methods were shown to be superior depending on the missingness proportion. As shown in Figure 4, this included the low-rank approximation–based imputation, predictive-mean matching–based multiple imputation, and expectation maximization–based multiple imputation for the 1% to 30% missingness proportions, while the low-rank approximation–based imputation and predictive-mean matching–based multiple imputation were superior for the 31% to 60% proportion, and only the low-rank approximation–based imputation was superior for proportions over 60%. These results are also shown in Table 4, which shows the sum of the pairwise comparison times with statistically small GAB for each missing-data handling method and missingness proportion. Listwise deletion, mean imputation, k-nearest neighbor–based imputation, expectation maximization–based multiple imputation, predictive-mean matching–based imputation, and low-rank approximation–based imputation achieved 15, 53, 2, 84, 91, and 99 as sums for the pairwise comparison times with statistically small GAB for 1% to 30% missingness proportions, respectively. The low-rank approximation–based imputation, predictive-mean matching–based multiple imputation, and expectation maximization–based multiple imputation were shown to be superior for these missingness proportions, with the low-rank approximation–based imputation revealing the maximum number (the predictive-mean matching–based and expectation maximization–based multiple imputations were also close to the maximum). The second and third rows of Table 4 show that the low-rank approximation–based imputation and predictive-mean matching–based multiple imputation were superior for the 30% to 60% missingness proportions, while only the low-rank approximation–based imputation was superior for over 60%.

**Table 4:** Sum of pairwise comparison times with statistically small GAB for each missing-data handling method and missingness proportion range.

Missingness proportion range	Listwise deletion	Mean imputation	k-nearest neighbor	Expectation–maximization	Predictive-mean matching	Low-rank approximation
1%-30%	15	53	2	84^a^	91^a^	99^a^
31%-60%	0	9	0	34	74^a^	75^a^
61%-80%	0	7	0	0	24	46^a^

^a^These methods had the best performance for the missingness proportion range.

## Discussion

### Principal Findings

The low-rank approximation–based imputation showed superior performance for 1% to 80% missingness proportions and has previously shown excellent performance with low-rank data sets [47]. In this context, low rank indicates that a data set can be approximated by a small subset of its singular vectors. Early studies [48,49] established strong theoretical guarantees about the perfect performance of low-rank approximation–based imputation for low-rank data sets without noise, with extensive research later supporting its superiority for low-rank data sets with noise [50-52]. These studies [48-52] suggest that the low-rank nature of the simulated data sets may be the primary reason that low-rank approximation–based imputation was shown to be superior in this study. In this regard, the low-rank property of the simulated data sets was investigated based on the chosen ranks for the low-rank approximation–based imputation to impute them. The rank of 13 was the maximum among the chosen ranks to impute all simulated data sets, while the maximum rank was much lower than the dimensions of the simulated data sets (ie, 884 × 49). It is therefore reasonable to assume that the low-rank nature of the simulated data sets is the primary reason that low-rank approximation–based imputation was shown to be superior.

Low-rank approximation–based imputation is also expected to perform well with other panel data sets comprising health behavior lifelogs, as previous studies [53,54] have verified that such data sets are generally low-rank. For instance, Eagle and Pentland [53] found that panel data sets comprising human behaviors were low-rank. They specifically proposed eigenbehaviors as principal components for panel data sets on human behaviors. The weighted sums of only 6 eigenbehaviors achieved more than 90% accuracy in reconstruction of a data set on the daily behaviors of 100 individuals for 400,000 hours. Furthermore, Saint Onge and Kreuger [54] found 7 distinct health lifestyle typologies for US adults in terms of 8 health behaviors, including sleep, physical activity, and alcohol intake. This result implied that panel data sets comprising health behaviors can be approximated by several typologies and are thus of a low-rank nature.

Both the expectation maximization–based and predictive-mean matching–based multiple imputations showed larger biases than the low-rank approximation–based imputation as the missingness proportion increased. Larger proportions increased the loss of information with missing values, which then increases uncertainty. Multiple imputation reflects such uncertainty in the standard errors of the estimates [14], with greater uncertainty resulting in larger standard errors for the estimates and larger coefficient biases [55].

In summary, the low-rank approximation–based imputation was the superior missing-data handling method for handling missing data when estimating a linear LWI with a panel data set comprising health behavior lifelogs, regardless of the missingness proportion.

### Future Research

Three future research issues can improve and expand on this research. The first involves validating generalizability of the current research to nonlinear LWIs (eg, functions with polynomial or interaction variables and logistic functions). New LWI development cases can aim to develop nonlinear LWIs that this study did not cover. Thus, additional research is needed to establish the validity of our findings in regard to nonlinear LWIs.

The second issue involves the need to identify which health behavior-related covariates (eg, age, gender, and BMI) can enhance the performance of missing-data handling for LWI estimation. While previous studies have already suggested several such covariates [56-58], additional covariates can enhance missing-data handling method performance. However, this study did not investigate these elements. Furthermore, few studies have identified covariates that can improve missing-data handling for panel data sets comprising health behavior lifelogs.

The third issue concerns the need to develop guidelines for predicting the size of bias in LWI coefficients for a certain missingness proportion of a given panel data set. In Figure 3, all missing-data handling methods showed increased coefficient biases as the missingness proportion increases. This suggests that missing-data handling methods can lead to large biases in LWI coefficients when missingness proportions are excessively large. Thus, a panel data set with a remarkably large missingness proportion requires careful attention to prevent excessively biased LWI coefficients. However, few previous studies have provided guidelines for predicting such biases according to the given missingness proportion. As shown in Figure 3, the low-rank approximation–based imputation exhibited linear growth in GAB as the missingness proportion increased. The slope of linear growth can be estimated through an experiment in which the change in GAB is calculated according to the unit change in the missingness proportion. The slope enables the prediction of GAB at a given missingness proportion. Such a guideline will help investigators decide whether the missingness proportion is acceptable for preventing highly biased coefficients of LWI. This requires additional research aimed at identifying relationships between biases and missingness proportions. Efforts are also needed to validate the generalizability of any guidelines.

### Conclusion

A panel data set comprising health behavior lifelogs will likely contain a large amount of missing data due to various events. These missing data can result in LWI coefficient biases. While there are various methods for handling missing data, few previous studies have set out to determine which are the most effective for reducing LWI coefficient biases. This study comparatively evaluated 6 representative missing-data handling methods by simulating an existing LWI development case. Results suggested that low-rank approximation–based imputation was superior for reducing biases when estimating a linear LWI with a panel data set composed of health behavior lifelogs. This finding is expected to contribute to the reduction of coefficient biases in new development cases where linear LWIs are estimated with panel data.

## Abbreviations

CVS: coefficient value set
GAB: grand-mean of absolute biases
LWI: lifelogs-based wellness index
MAB: mean of absolute biases
